# Definition of Human Epitopes Recognized in Tetanus Toxoid and Development of an Assay Strategy to Detect *Ex Vivo *Tetanus CD4^+^ T Cell Responses

**DOI:** 10.1371/journal.pone.0169086

**Published:** 2017-01-12

**Authors:** Ricardo da Silva Antunes, Sinu Paul, John Sidney, Daniela Weiskopf, Jennifer M. Dan, Elizabeth Phillips, Simon Mallal, Shane Crotty, Alessandro Sette, Cecilia S. Lindestam Arlehamn

**Affiliations:** 1 La Jolla Institute for Allergy and Immunology, La Jolla, California, United Ststes of America; 2 Institute for Immunology and Infectious Diseases, Murdoch University, Perth, Western Australia, Australia; Tulane University School of Public Health and Tropical Medicine, UNITED STATES

## Abstract

Despite widespread uses of tetanus toxoid (TT) as a vaccine, model antigen and protein carrier, TT epitopes have been poorly characterized. Herein we defined the human CD4^+^ T cell epitope repertoire by reevaluation of previously described epitopes and evaluation of those derived from prediction of HLA Class II binding. Forty-seven epitopes were identified following *in vitro* TT stimulation, with 28 epitopes accounting for 90% of the total response. Despite this diverse range of epitopes, individual responses were associated with only a few immunodominant epitopes, with each donor responding on average to 3 epitopes. For the top 14 epitopes, HLA restriction could be inferred based on HLA typing of the responding donors. HLA binding predictions re-identified the vast majority of known epitopes, and identified 24 additional novel epitopes. With these epitopes, we created a TT epitope pool, which allowed us to characterize TT responses directly *ex vivo* using a cytokine-independent Activation Induced Marker (AIM) assay. These TT responses were highly Th1 or Th2 polarized, which was dependent upon the original priming vaccine, either the cellular DTwP or acellular DTaP formulation. This polarization remained despite the original priming having occurred decades past and a recent booster immunization with a reduced acellular vaccine formulation. While TT responses following booster vaccination were not durably increased in magnitude, they were associated with a relative expansion of CD4^+^ effector memory T cells.

## Introduction

Infection with *Clostridium tetani*, a ubiquitous bacterium in soil and the environment can lead to tetanus, a disease characterized by progressive muscle spasms and up to a 10% mortality rate [1]. The disease results from production of bacterial toxin. A tetanus vaccine, based on inactivated tetanus toxin known as tetanus toxoid (TT), has been available in the USA since the 1940s and has led to a 95% decrease in disease incidence [2]. After 1995–1996 immunization started to be performed with TT as a component of acellular vaccines such as the current licensed DTaP and Tdap which contain difteria, tetanus and pertussis vaccines, further increasing its widespread use in the USA population and more generally worldwide [3, 4]. Five doses are recommended in childhood, followed by boosters every ten years. However it is common practice to administer a TT booster to puncture wound patients when the immunization status is uncertain.

Key components of a successful vaccination include development and maintenance of sufficient titers of neutralizing antibodies [5], and CD4^+^ T helper (Th) responses to the vaccine [6]. Indeed, the TT antigen has been recognized as a powerful antigen for Th responses with intrinsic adjuvant properties [7, 8]. It has also been used as model antigen/carrier for many basic immunological studies and as a carrier for polysaccharide based vaccines, where the antibody epitopes are devoid of Th cell activity, such as Meningococcal Groups C and Y and Haemophilus b [9, 10].

Surprisingly, in light of its prominent role in vaccinology and basic immunology alike, the exact nature of the human Class II restricted TT epitopes has not been thoroughly analyzed despite several studies having defined epitopes [11–16]. Several studies have delineated some of the epitopes with earlier studies utilizing T cell clones, thus raising questions about how representative these epitopes might be of those recognized in the general population [11, 14]. A few landmark studies [12, 13, 16] defined additional epitopes recognized in proliferation assays, however, these assays did not allow for further phenotyping of the responding cells. Indeed, *ex vivo* analysis of human TT responses have been limited, and hampered by a lack of well-defined epitopes and assay systems.

Previous studies of epitope biology, in the contexts of erythropoietin (EPO) and the timothy grass (Phl p) allergens, identified HLA Class II binding promiscuity (capacity to bind multiple HLA class II molecules) as a good predictor of immunogenicity [17–19]. Based on these initial studies, HLA Class II binding promiscuity has been successfully utilized to identify a significant fraction of the antigen specific response in several different indications, including common allergens [20] cockroach allergens [21] novel Timothy grass allergens [22] and *Mycobacterium tuberculosis* [23]. Additionally, previous work from our laboratory demonstrated that sequential lyophilization can be used to develop pools of large number of epitopes (Megapools) which avoids the toxicity associated with solubilization of each individual epitope [24].

Here, we developed a TT-specific megapool consisting of previously identified epitopes, and 24 new epitopes, based on predicted HLA binding capacity. Independently, we recently reported the development of a new Activation Induced Marker (AIM) assay, which allows detecting *ex vivo* responses of human PBMC [25]. Accordingly, we demonstrate that our TT-specific megapool, in conjunction with the AIM assay, detects *ex vivo* TT-specific human CD4^+^ T cells and associated memory Th subsets.

## Materials and Methods

### Study subjects

We recruited of 36 healthy adults from San Diego, USA (S1 Table). All participants provided written informed consent for participation in this cross-sectional study and clinical medical history was collected and evaluated. Individuals, who have been diagnosed with *Clostridium tetani* infection at any given time in their life, were excluded. A subset of these donors, either originally vaccinated with DTwP or DTaP in infancy, received recently a booster vaccination with Tdap. The remaining donors had not, to the best of our knowledge, received recent booster immunizations in the last 4 years. This study was performed with approvals from the Institutional Review Board at La Jolla Institute for Allergy and Immunology (protocols; VD-101-0513 and VD-059-0813).

### Peptides

Peptides were derived from the *Clostridium tetani* toxin (TT) sequence (Acc. No. P04958) by combining two different selection processes. A first selection approach identified 40 peptides by searching the Immune Epitope Database (IEDB) [26] for "Human Class II responses to tetanus” and using the immunobrowser tool [27] to select the top 40 peptides tested in at least 8 different donors and positive in 3 or more donors. Individual peptides longer than 20 residues were parsed into 2 or 3 overlapping 15- or 16-mers spanning the longer sequence. Accordingly, this resulted in a total of 44 peptides from the IEDB selection process.

In the second selection approach, we selected peptides based on the epitope candidate prediction protocol described previously [28]. As per this protocol, a set of 261 15-mer peptides, overlapping by 10 residues, spanning the complete TT sequence was generated. The binding affinity of these peptides to seven common HLA DR alleles (3 DRB1 and 4 DRB3/4/5) was predicted using IEDB recommended method of the IEDB class II binding prediction tool (http://tools.iedb.org/mhcii/). The median of the predicted IEDB consensus percentile scores of the seven alleles was estimated for each peptide and the peptides with the median percentile score < 20.0 were selected as the top predicted epitope candidates–a total of 124 peptides. We next determined the sequence overlap of these 124 peptides with the peptides identified from the IEDB (above). Of the 44 peptides reported in the IEDB, 37 (84%) peptides nested (or largely overlapped) with one or more of the predicted 124 epitope candidates. Therefore, the second selection approach identified 81 predicted peptides that were unique from the IEDB panel. Accordingly, from both selection approaches a total of 125 peptides were synthesized as crude material on a small (1mg) scale by A and A (San Diego, California) and tested as described below. The 125 peptides are listed in S2 Table.

### PBMC isolation and culture

PBMC (peripheral blood mononuclear cells) were isolated from collected blood or leukapheresis by density gradient centrifugation, according to the manufacturer’s instructions. Cells were cryopreserved in liquid nitrogen suspended in FBS containing 10% (vol/vol) DMSO. For PBMCs *in vitro* expansion, 2 x 10^6^ cells/ml were cultured in the presence of 5 μg/ml Tetanus Toxoid (TT) (List Biological Laboratories Inc., Campbell, CA) in RPMI media (Omega Scientific) supplemented with 5% human AB serum (Gemini Bioscience), GlutaMAX (Gibco), and penicillin/streptomycin (Omega Scientific). Every 3 days, 10 U/ml IL-2 in media was added to the cultures.

### ELISPOT, ICS and AIM assays

After 14 days of culture with TT, the response to peptides, peptide pools, or whole antigens was measured by IFNγ and IL-5 dual ELISPOT as previously described [20]. Briefly, cells were harvested and plated at a density of 5x10^4^ cells/well with either individual peptides (10 μg/ml), peptide pools (5 μg/ml) or TT antigen (5 μg/ml). PHA (10 μg/ml) was used as a positive control and medium containing 0.13% DMSO (corresponding to the percentage of DMSO in the pools/peptides) was used as a negative control. ELISPOT plates were then incubated for 24 h. Consistent with previous studies [22], to be considered positive, a peptide pool response had to match all of three different criteria. These three criteria were to elicit at least 100 spot-forming cells (SFC) per 10^6^ PBMC, *p* ≤ 0.05 by Student’s t-test or by a Poisson distribution test, and stimulation index ≥ 2. Criteria for peptide positivity were identical except with a threshold of 20 SFCs per 10^6^ PBMC was utilized. Cumulative SFC was calculated by sorting the epitopes according to their total SFC or according to the consensus percentile for binding prediction, and then calculating the cumulative sum of responses for each peptide or consensus percentile from highest to lowest. The percentage of cumulative response was calculated by dividing each individual value with the total.

For the Activation Induced Marker (AIM) assay we used a protocol described elsewhere [25]. This assay detects cells that are activated as a result of antigen specific stimulation by staining antigen-experienced CD4^+^ T cells for TCR-dependent upregulation of OX40 and CD25 and/or PD-L1 after an optimal time of 18–24 h of culture. This method exhibits increased sensitivity and is one of the most robust methods for identification of Ag-specific cells in humans. Briefly, cryopreserved PBMCs were thawed and 1x10^6^ cells/condition were immediately cultured together with TT peptide pools (2 μg/ml), Dengue virus peptide pool (1 μg/ml), TT (5 μg/ml), or PHA (10 μg/ml; Roche) as a positive control in 5% human serum (Gemini Bioproducts) for 24 hours. To determine the memory phenotype of responding T cells staining for CD45RA and CCR7 were performed.

For intracellular cytokine staining (ICS), PBMCs were incubated with TT peptide pool for 2h. After 2h, BFA (1ug/ml [BD Bioscience, San Diego, CA]) was added for an additional 4h. Cells were then washed, stained for extracellular markers for 30 min, and then washed, fixed with 4% paraformaldehyde, permeabilized with 0.5% saponin (Sigma) and stained for intracellular IFNγ and IL-4. A combination of PMA and Ionomycin (1 μg/ml) was used as positive control. Samples for both AIM and ICS were acquired on a BD LSRII Flow Cytometer and analyzed using FlowJo X Software. All flow cytometry mAb reagents are shown in S3 Table.

### HLA typing and inferred restriction

HLA typing was performed either at LJI or by an American Society for Histocompatibility and Immunogenetics-accredited laboratory at Murdoch University. HLA typing was performed for Class I (HLA A; B; C) and Class II (DQA1; DQB1, DRB1 3,4,5; DPB1) using locus-specific PCR amplification on genomic DNA. Primers used for amplification employed patient-specific barcoded primers. Amplified products were quantitated and pooled by subject and up to 48 subjects were pooled. An unindexed (454 8-lane runs) or indexed (8 indexed MiSeq runs) library was then quantitated using Kappa universal QPCR library quantification kits. Sequencing was performed using either a Roche 454 FLX+ sequencer with titanium chemistry or an Illumina MiSeq using a 2 x 300 paired-end chemistry. Reads were quality-filtered and passed through a proprietary allele calling algorithm and analysis pipeline using the latest IMGT HLA allele database as a reference. Potential HLA-epitope restriction odds ratios and relative frequencies were calculated using the RATE program [29].

## Results

### Repertoire and immunodominance of tetanus-derived human T cell epitopes

To define the patterns of T cell responses and identify TT derived T cell epitopes, we utilized a strategy we successfully applied to several other systems [17, 21–23], relying on *in vitro* stimulation of PBMCs for 14 days with whole antigen. Accordingly, PBMCs were incubated with whole TT antigen for 14 days in the presence of IL-2, followed by testing 13 pools of approximately 10 peptides each. Positive pools were deconvoluted to identify individual epitopes.

Overall, T cell responses were analyzed using this strategy for a total of 20 individuals (S1 Table). Of these donors, seven were boosted within six months prior the donation while the remaining 13 were not, to the best of our knowledge, recently boosted. Positive responses were detected for a total of 47 epitopes. There was no difference when comparing epitope recognition between donors that were primed with DTaP versus DTwP (S2 Table). The sequence, amino acid position and length of these 47 epitopes, together with the total response in terms of SFC/10^6^ detected and frequency of associated positive responses are listed in S2 Table.

**Fig 1:**
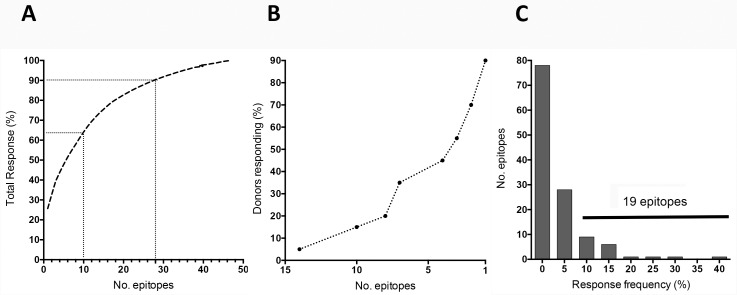
Response to tetanus toxoid is broad but characterized by a high degree of immunodominance. (A) Epitopes ranked on the basis of magnitude of response. Dotted lines indicate the top 10 (65% of total response) and top 28 (90% of total response) epitopes. (B) Breadth of response indicated by proportion of donors who respond to the specified number of epitopes. (C) Epitopes plotted as a function of the response frequency. Black line indicates the 19 epitopes that are recognized by 10% or more.

The top 28 epitopes (S2 Table) accounted for 90%, and the top 10 epitopes for 65%, of the total magnitude of the detected responses (Fig 1A). As shown in Fig 1B, over 90% of donors recognized at least one epitope, and 50% of the donors responded to ≥3 epitopes. Thus, TT responses are associated with a significant degree of immunodominance, in that the responses are associated, in each individual, with relatively few epitopes.

Conversely, a total of 19 epitopes were recognized in at least 2 out of the 20 donors tested, corresponding to a frequency of recognition of 10% (S2 Table). Only four epitopes were recognized in 20% or more of the donors (4/20 positives). Thus, which particular epitopes are going to be immunodominant is highly variable from one individual to the next (Fig 1C).

### Inferred HLA restriction of dominant epitopes

To correlate HLA types with T cell responses and to facilitate the design of tetramer reagents to detect antigen-specific T cells, we used the RATE program [29] to calculate the relative frequency and significance of association between all the epitopes/regions and HLA alleles (or combinations thereof) expressed in responding donors.

This analysis allowed inferring potential restrictions for the 14 of the main epitopes. All of these 14 restrictions were promiscuous restrictions (the epitope was inferred to be restricted by multiple HLAs), thus confirming and extending previous results that suggested that dominant epitopes are often associated with promiscuous HLA restriction [17, 23]. It should be emphasized that these inferred alleles are meant to restrict the potential choices for the most likely restricting elements, and that further experimentation is required to conclusively assign restriction. S4 Table lists the inferred restrictions for each of these epitopes, and details the number and combinations of donors that responded (R+) or did not respond (R-) to a given epitope, and the number of donors expressing (A+) or not expressing (A-) a given HLA(s) Accordingly, for example, the TT.152 epitope (AMLTNLIIFGPGPVLNKNEV), 100% (8/8) of the responders express HLA DRB1*15:01, DRB1*07:01 or DRB5*01:01, while only 9% of the non-responders expressed the same HLAs (p<0.001).

### Comparison of known epitopes to predicted epitopes

As stated above, the set of peptides tested were either already described in the literature as being recognized by human CD4^+^ T cells, and/or predicted to bind HLA molecules by a recently described prediction protocol [28]. Interestingly, 37/44 (84%) of the previously described epitopes were contained within the predicted set, thus validating in an independent setting the previous results.

**Fig 2:**
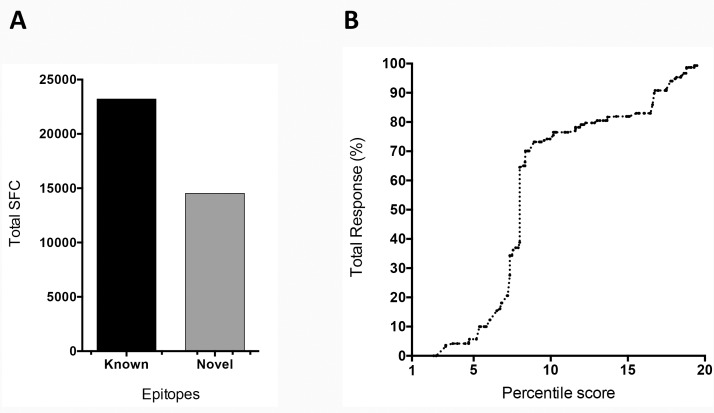
Bioinformatics predictions efficiently identify the preponderance of the tetanus toxin response. (A) Total SFC detected against known (black bar) and novel (grey bar) epitopes. (B) Percentage of the total response captured by the indicated percentile score from peptide binding predictions.

We further found that 38.5% of the total response, was accounted for by 24 of the novel identified epitopes (Fig 2A), thus underlining that the present study led to the identification of several new epitopes and a more comprehensive response coverage. We further analyzed the performance of the predictive method by plotting the percentage total response as a function of the percentile score used for prediction (Fig 2B). Since little response is seen with epitopes beyond the 15th percentile, we conclude from this analysis that the prediction was exhaustive and truly represent a comprehensive account of TT specific responses.

### Similar magnitude of T cell responses following booster vaccination

**Fig 3:**
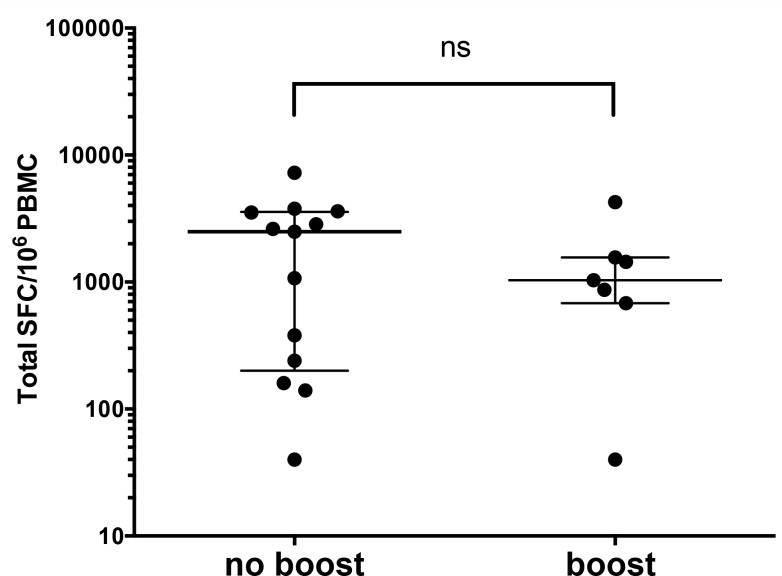
Equivalent levels of tetanus-specific T cell responses in recently boosted individuals as compared to non-recently boosted individuals. Sum of T cell responses for all DTaP antigens for non-boosted donors (no boost) or donors boosted with Tdap 0.5–6 months ago (boost). Each individual data point represents one donor, median ± interquartile range is shown. ns: no significant difference by two-tailed Mann-Whitney test.

As mentioned above, some of the PBMC donations analyzed were derived from recent Tdap boosted donors. Accordingly we plotted separately responses from donors boosted with the Tdap vaccine within the previous 6 months, as detailed in S1 Table, in comparison with donors that were not (to the best of our knowledge) boosted within the last six months. Unexpectedly, no significant difference in the level of T cell responses was noted between the recently boosted and not recently boosted donor cohorts (Fig 3).

### Differential polarization of TT cell responses as a function of the original DTwP and DTaP priming

The acellular DTaP vaccination has been reported to induce greater responses of the Th2 subset compared to the cellular DTwP formulation, and this Th2 bias is maintained in 4-6-year-olds boosted with Tdap, and it extends to the TT component [6, 30, 31]. Here we sought to examine whether this bias in TT responses is maintained into adolescence and adulthood like we previously reported for pertussis (PT) responses [32]. As shown in S1 Table, 10 of our donors were originally primed with DTaP and 26 with DTwP. However since the use of DTwP was discontinued in the USA approximately in 1995–1996, all donors were subsequently boosted in adolescence and adulthood with Tdap.

**Fig 4:**
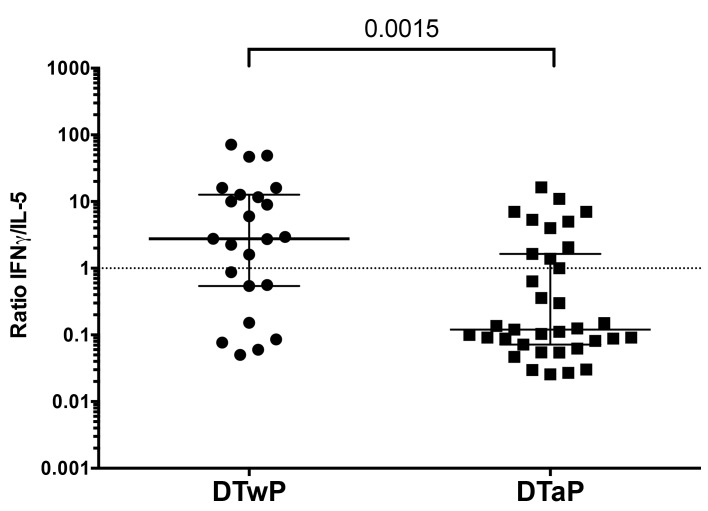
Differential polarization of T cell responses as a function of the original vaccine type used for childhood vaccination. IFNγ and IL-5 responses were measured by dual color ELISPOT assays. Each data point represents the ratio of IFNγ/IL-5 responses of each positive individual peptide from all the reactive donors. Median ± interquartile range for donors originally primed with DTwP or DTaP vaccine is represented. Two-tailed Mann-Whitney test.

We examined the polarization of T cell responses based on ratio of IFNγ /IL-5 production per epitope tested (Fig 4) as a function of whether the donor was originally primed with DTaP or DTwP. The dominant cytokine secreted upon stimulation with the individual epitopes was IFNγ for individuals who had first received the cellular DTwP, (median 2.76, IQR 12.11). For individuals who had first received DTaP, the dominant cytokine was IL-5 (median 0.12, IQR 1.57). These data demonstrate that the significant difference in Th1/Th2 polarization of TT responses as a function of original childhood priming is still prominent decades later and even following identical booster with the Tdap vaccine.

### A TT-megapool and AIM assay allow detection of human TT-specific CD4^+^ T cells *ex vivo*

We recently reported a strategy to generate pools of large numbers of potential epitopes for use in cellular assays and avoid toxicity associated with high amount of DMSO required to achieve solubility of such large numbers of peptides [24, 33]. Herein we applied this “megapool” strategy to the detection of TT specific responses.

**Fig 5:**
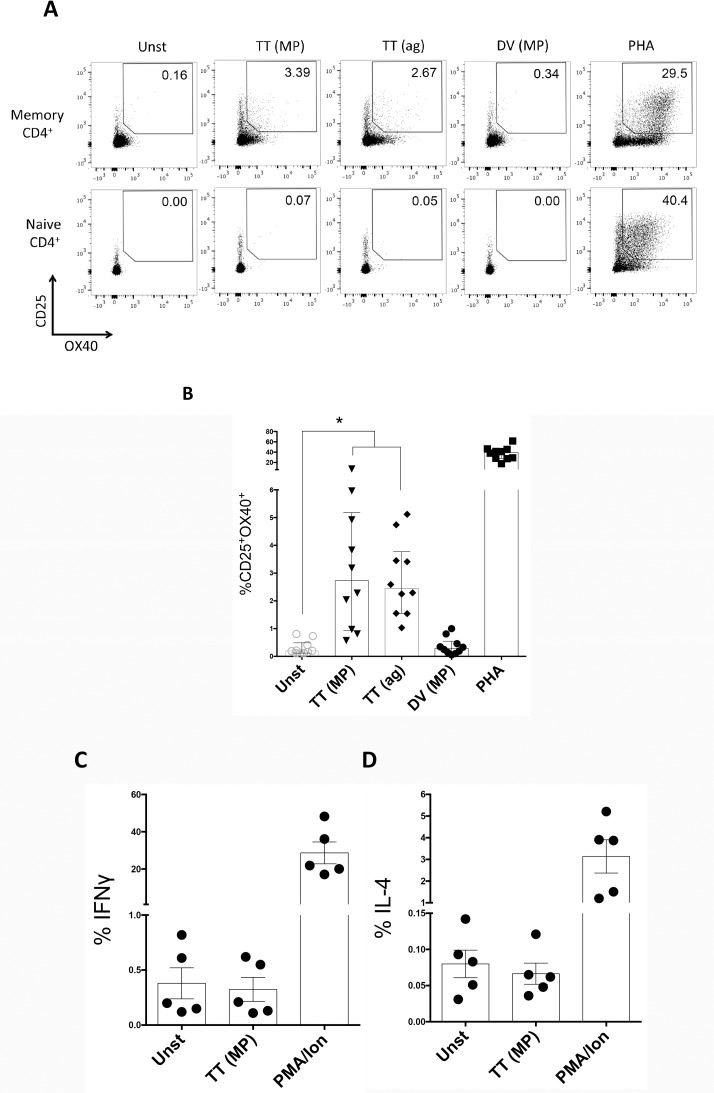
AIM assay detects tetanus-specific CD4^+^ T cells. (A) Representative flow cytometry plots of CD25^+^OX40^+^ upregulation by CD4^+^ T cells in naïve (CD45RA^+^CCR7^+^; lower panel) and antigen-experienced memory (excluding naïve cells; upper panel) cells after stimulation with TT-megapool [TT (MP)], Tetanus Toxoid [TT (ag)], Dengue virus-megapool [DV (MP)], or PHA as a positive control. (B) Median CD25^+^OX40^+^ expression by CD4^+^ memory T cells after 24 hours. Each dot represents one donor originally primed with DTwP vaccine and not recently boosted. Kruskal-Wallis multiple comparison test, *, *p*<0.05. (C and D) Percentage IFNγ- (C) or IL-4-producing (D) CD4^+^ memory T cells in response to TT megapool, or PMA/Ion.

A TT-specific megapool including all potential epitopes was prepared since peptides not found as positive in the current analysis might nevertheless be positive in donors with a different HLA representation. This megapool was tested in the direct *ex vivo* AIM assay (Fig 5). AIM is a cytokine-independent method of detecting antigen-specific CD4 T cells based on TCR-dependent upregulation of CD25, OX40, and/or PD-L1 [25]. The results for one representative donor are shown in Fig 5A, and a compilation of results for donors originally primed with DTwP donors and not recently boosted, is shown in Fig 5B. These donors were largely representative of an independent set of donors, since PBMC of only 1 out of 10 donors were utilized in the experiment described in the previous sections. Significant TT-specific responses are seen by AIM assay with 0.57% to 7.37% of the cells being positive (median 2.73, IQR 4.25). Equivalent or somewhat lower responses are detected when using whole TT protein as the stimulating antigen (median 2.44, IQR 2.23).

The specificity of this assay is further confirmed by absent or lower reactivity against a control megapool (composed of dengue virus Class II restricted epitopes) along with the DMSO stimulation control (Fig 5B). Furthermore, in the same experiments we also utilized in parallel conventional identification of antigen-specific cells by intracellular cytokine staining (ICS), upon TT-specific megapool stimulation. However, we could not detect consistently significant amounts of IFNγ or IL-4 (Fig 5C and 5D), thus emphasizing the value of the combined megapool/AIM approach assay to identify antigen-specific cells. This data validated the use of the TT-megapool for use in direct *ex vivo* determinations utilizing the AIM assay.

### Booster immunization does not induce higher antigen specific responses *ex vivo* but modulates T cell memory subsets

**Fig 6:**
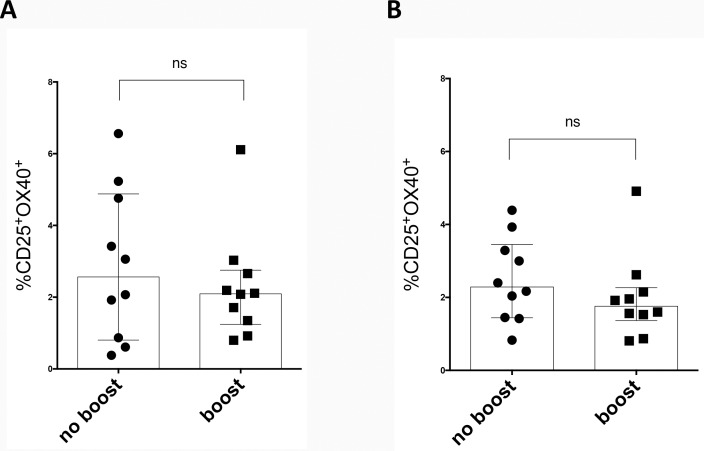
Similar levels of TT-specific CD4^+^ memory T cells after boosting as detected by the AIM assay. CD25^+^OX40^+^ expression by CD4^+^ memory T cells after 24 hours of culture for donors originally primed with DTwP vaccine not boosted (no boost) or 0.5–6 months post Tdap boost (boost) after TT-megapool (A) or TT (B) stimulation. Each dot represents one donor. Median ± interquartile range is shown. ns: no significant difference by two-tailed Mann-Whitney test

**Fig 7:**
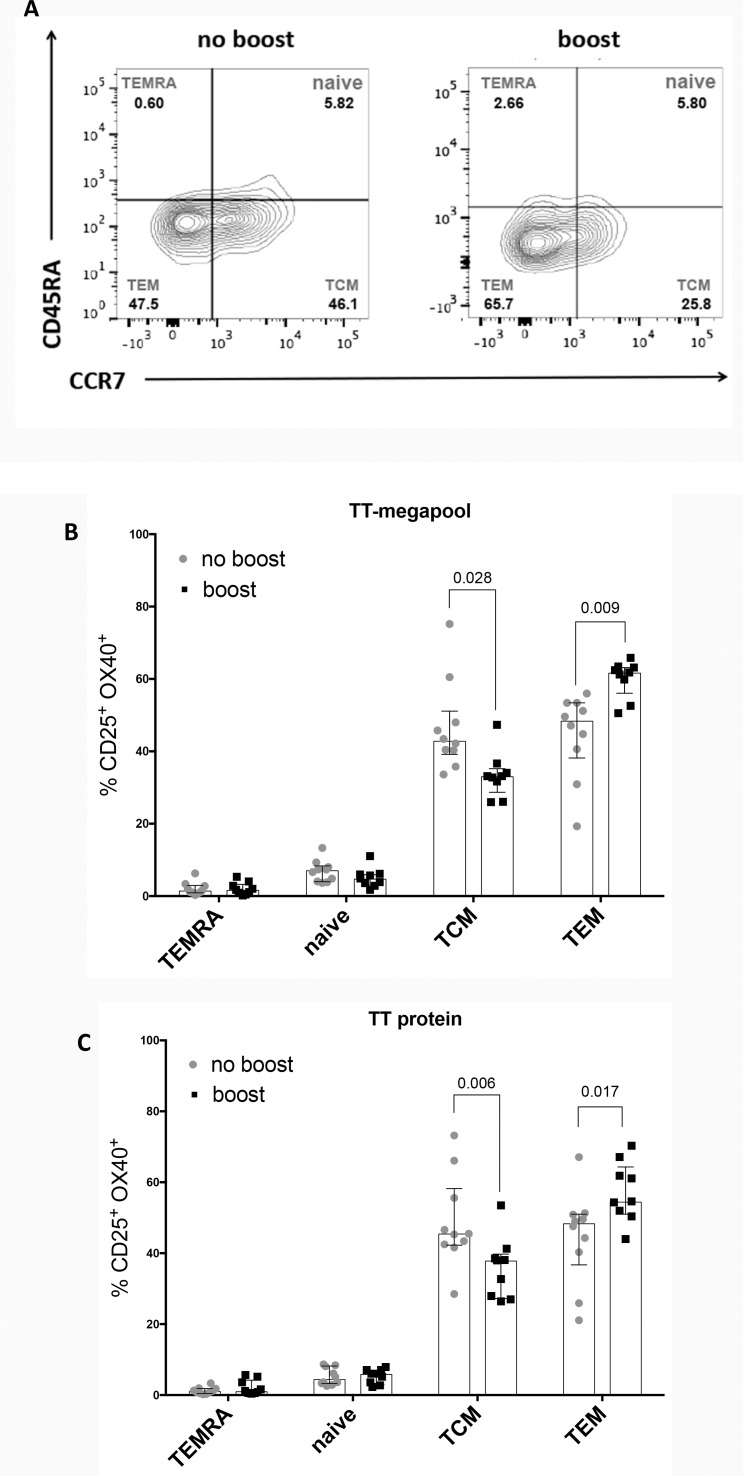
AIM Assay detects tetanus-specific CD4^+^ effector memory T cell expansion. (A) Representative flow cytometry plots of CD4^+^CD25^+^OX40^+^ T cells gated on CD45RA and CCR7 expression for central memory (CD45RA^-^ CCR7^+^), effector memory (CD45RA^-^ CCR7^-^), naïve (CD45RA^+^ CCR7^+^), and TEMRA (CD45RA^+^ CCR7^-^) cells after 24 hours of TT-megapool stimulation for a donor originally primed with DTwP vaccine and not boosted (no boost) or after Tdap booster vaccination (boost). The CD4^+^ cell response to TT-megapool (B) or TT whole protein (C) shows a significant increase after boosting of the effector memory population (*p* = 0.009 and *p* = 0.017 respectively) and concomitant decrease of the central memory population (*p* = 0.028 and *p* = 0.006 respectively). Two-tailed Mann-Whitney test. Median ± interquartile range is shown. Each dot represents one donor.

In the same series of experiments we compared 10 donors boosted with the Tdap, as detailed in S1 Table, to individuals not boosted within the last six months. Comparable with the results obtained following *in vitro* re-stimulation, the AIM assay showed no differences in TT-specific memory CD4^+^ T cells between Tdap boosted and not boosted donor cohorts using either a TT-megapool (Fig 6A) or TT protein (Fig 6B). However, upon booster vaccination, we observed modulation of the central (TCM, CD45RA^-^CCR7^+^) and effector (TEM, CD45RA^-^CCR7^-^) memory subsets. Specifically (Fig 7A and 7B), we observed a significant increase of the fraction of TT-specific response associated with the CD4^+^ TEM subset and concomitant decrease of the CD4^+^ TCM population. Similar results were observed for tetanus toxoid antigen stimulation with whole protein (Fig 7C). These results are consistent with relatively recent *in vivo* activation of the TT-specific CD4^+^ T cells.

## Discussion

In the present study we investigated human TT-derived epitopes recognized in humans, and defined associated patterns of breadth of response, immunodominance, relationship to recent boosting and polarization of responses as a function of the original childhood vaccination. We further show that a TT-megapool can be used in conjunction with the newly described AIM method to detect and study TT-specific CD4^+^ T cell responses directly *ex vivo*.

It is well appreciated that human CD4^+^ T cell responses are very heterogeneous, and that depending on the specific antigen studied, ten or even hundreds of epitopes are recognized at the individual and population level. This remarkable level of heterogeneity and breadth must be balanced in the context of the phenomenon of immunodominance, referring to the common observation that few epitopes dominate in a given individual the majority of responses. Our investigation emphasizes how TT responses are associated with a significant degree of immunodominance, in that the responses are associated, in each individual, with relatively few epitopes. However, which particular epitopes are going to be immunodominant is highly variable from one individual to the next.

The peptides tested were either already described in the literature as being recognized by human T cell responses, and/or predicted to bind HLA molecules by a recently described population approach [28]. Interestingly, nearly all of the previously described epitopes were contained within the predicted set, thus validating in an independent setting the results of the previous studies [20–23]. Conversely, no significant responses were detected against the “universal” TT epitope corresponding to residues 830–843 [11, 15]. Since the original data regarding this epitope was obtained with T cell clones following repeated *in vitro* stimulation, and much of the subsequent studies were performed using the epitope as an *in vitro* stimulus, we conclude that this epitope is probably a relatively rare subdominant specificity, which can be expanded by direct stimulation with the epitope.

We furthermore observe that a previously described protocol [28] to predict candidate T cell epitopes at the population level identified a subset of 124 peptides out of 261 total overlapping 15-mers as potential epitopes. The extremely high number predicted HLA class II binders identified by the prediction analysis (124/261, 48%) in the toxin sequence might reflect a TT bias towards high binding peptide content, which in turn might explain its strong immunogenicity for humans. In identifying 24 novel epitopes our study further validated the prediction method, demonstrating the importance of HLA binding as a necessary but not sufficient feature of Class II restricted epitopes.

Surprisingly, there was no significant difference in the magnitude of the antigen-experienced TT-specific CD4^+^ T cell response between donors boosted within the last six months versus not-recently-boosted donors using two independent methodology assays. This observation is consistent with the study by Cellerai *et al*., which reported peak CD4^+^ T cell expansion 11 days after priming, returning to baseline after two months [34]. This narrow window in which the TT-specific CD4^+^ T cell responses are elevated, might be related to the high rate of tetanus vaccination in the general USA population, associated with already fairly elevated TT-specific CD4^+^ T cell population, resulting in only transient boosting of TT-specific CD4^+^ T cells. In fact, in our limited dataset, we detected a trend towards *lower* TT-specific CD4^+^ T cells following booster vaccination, suggesting that overly frequent booster immunization may be associated with lower responses [35].

In the mid-1990 the use of whole cell pertussis vaccination (DTwP) was phased out in favor of the less reactive acellular form of the vaccine (DTaP). Previous studies highlighted how this different vaccine was associated, in the newborn and up to 9 years of age, with a polarization switch towards Th2 responses. The DTwP vaccine induced a predominant Th1 response, while the DTaP vaccine induced a predominant Th2 response [6, 30, 31, 36]. This response bias extended to the TT, which is a component of the trivalent tetanus diphtheria and pertussis vaccines commonly utilized. Our results show that this significant difference in Th1/Th2 polarization of TT responses as a function of original DTaP/DTwP priming is maintained for years, and is still prominent even following identical booster with the Tdap vaccine and suggests that it might involve enactment of specific molecular programs in memory T cells. These results parallel our previous findings for PT responses in young adults and adults [32] and validates the use of TT-megapool as a tool to detect TT-specific responses and to better understand how the polarized persistence of different vaccines, stubbornly maintained for decades, might impact the protective efficacy.

Hereby, we also performed sequential lyophilization to develop pools of large number of epitopes (Megapools) [24]. By the use of this protocol pools of hundreds of epitopes can be formulated to maintain solubility of each component at sufficiently high concentrations, enabling testing in low and non-toxic solvent concentrations. The present results validate previous observations in the case of dust mite allergens, DENV and tuberculosis infection that have shown that the megapool approach allows detecting responses directly *ex vivo* [24, 33, 37, 38].

Characterization of antigen-specific CD4^+^ T cells using well-defined antigenic stimuli is highly desirable in human translational immunology. We used a TT megapool, which, in combination with the AIM assay, permitted reproducible detection of TT-specific CD4^+^ T cells in healthy blood donors. *Ex vivo* phenotypic analysis of defined T cell populations is critical, since *in vitro* culture may alter composition and functional properties of the populations of interest. Subsequent phenotypic characterization showed a significant difference in the level of T cell effector *versus* central memory compartments between the recently boosted and not recently boosted donor cohorts.

Taken together, these results validate the combination of a megapool approach and the AIM assay as a general tool to study TT-specific responses in humans.

## Supporting Information

S1 Table Characteristics of the donor population in the study. Age, priming vaccination, booster vaccination, time since last boost, and the number of samples taken for each cohort is shown. Age is expressed as the mean ± standard error for the entire group.
(XLSX)

S2 Table Peptides tested and recognized epitopes. Peptide sequence, start position, peptide set, no. of donors tested, no. of donors positive for IFNγ / IL-5 or any of the two cytokines, as well as individual donor responses. Table is sorted according to Total SFC.
(XLSX)

S3 Table List of anti-human antibodies for surface staining included in this study. (XLSX)

S4 Table Bioinformatics inferred epitope restrictions. Shown are the HLA restriction of the dominant epitopes represented by the epitope sequence, start position, restricting alleles, number of donors that responded (R+) or did not respond (R-) to a given peptide, number of donors expressing (A+) or not expressing (A-) given HLAs, the response frequency, odds ratio and the p-value associated associated with its significance estimated by Fisher's exact test.
(XLSX)

## Abbreviations

AIM: Activation Induced Marker
DTaP: Diphtheria & Tetanus toxoids and acellular Pertussis vaccine
DTwP: Diphtheria & Tetanus toxoids and whole-cell Pertussis vaccine
IEDB: Immune Epitope Database
SFC: spot-forming cell
TCM: central memory T cells
TEM: effector memory T cells
Tdap: Tetanus & reduced diphtheria toxoids and acellular pertussis boost vaccine
TT: Tetanus toxoid
PT: Pertussis
