# Correction: Definition of Human Epitopes Recognized in Tetanus Toxoid and Development of an Assay Strategy to Detect *Ex Vivo* Tetanus CD4^+^ T Cell Responses

**DOI:** 10.1371/journal.pone.0193382

**Published:** 2018-02-20

**Authors:** Ricardo da Silva Antunes, Sinu Paul, John Sidney, Daniela Weiskopf, Jennifer M. Dan, Elizabeth Phillips, Simon Mallal, Shane Crotty, Alessandro Sette, Cecilia S. Lindestam Arlehamn

The affiliation for the eighth author is incorrect. Shane Crotty is not affiliated with #2 but with #1 La Jolla Institute for Allergy and Immunology, La Jolla, California, United States of America.
